# Anti-Inflammatory Effect of a Peptide Derived from the Synbiotics, Fermented *Cudrania tricuspidata* with *Lactobacillus gasseri*, on Inflammatory Bowel Disease

**DOI:** 10.1155/2020/3572809

**Published:** 2020-07-04

**Authors:** Jimyeong Ha, Hyemin Oh, Nam Su Oh, Yeongeun Seo, Joohyun Kang, Min Hee Park, Kyung Su Kim, Shin Ho Kang, Yohan Yoon

**Affiliations:** ^1^Risk Analysis Research Center, Sookmyung Women's University, Seoul 04310, Republic of Korea; ^2^Department of Food and Nutrition, Sookmyung Women's University, Seoul 04310, Republic of Korea; ^3^Department of Food Science and Biotechnology, Korea University, Sejong 30019, Republic of Korea; ^4^R&D Center, Seoul Dairy Cooperative, Ansan 15407, Republic of Korea

## Abstract

The objective of this study was to evaluate the effects of peptides derived from synbiotics on improving inflammatory bowel disease (IBD). Five-week-old male C57BL/6 mice were administered with dextran sulfate sodium (DSS) via drinking water for seven days to induce IBD (IBD group). The mice in the IBD group were orally administered with PBS (IBD-PBS-positive control), *Lactobacillus gasseri* 505 (IBD-Pro), fermented powder of CT extract with *L. gasseri* 505 (IBD-Syn), *β*-casein: LSQSKVLPVPQKAVPYPQRDMP (IBD-Pep 1), or *α*_s2_-casein: VYQHQKAMKPWIQPKTKVIPYVRYL (IBD-Pep 2) (both peptides are present in the synbiotics) for four more days while inducing IBD. To confirm IBD induction, the weights of the animals and the disease activity index (DAI) scores were evaluated once every two days. Following treatment of probiotics, synbiotics, or peptides for 11 days, the mice were sacrificed. The length of the small and large intestines was measured. The expression of the proinflammatory cytokines IL-1*β*, IL-6, TNF-*α*, and COX-2 in the large intestine was measured. Large intestine tissue was fixed in 10% formalin and stained with hematoxylin and eosin for histopathological analysis. The body weights decreased and DAI scores increased in the IBD group, but the DAI scores were lower in the IBD-Pep 2 group than those in the IBD group treated with PBS, Pro, Syn, or Pep 1. The lengths of the small and large intestines were shorter in the IBD group than in the group without IBD, and the expression levels of the proinflammatory cytokines were lower (*p* < 0.05) in the IBD-Pep 2 group than those in the IBD-PBS-positive control group. In addition, histopathological analysis showed that IBD was ameliorated in the Pep 2-treated group. These results indicate that Pep 2 derived from *α*_s2_-casein was effective in alleviating IBD-associated inflammation. Thus, we showed that these peptides can alleviate inflammation in IBD.

## 1. Introduction

Inflammatory bowel disease (IBD) is a chronic inflammation in the intestine, and the number of IBD patients has increased by 12 to 14.5 times, especially for pediatric patients [1, 2]. IBD is caused by abnormal immune responses and dysbiosis of intestinal bacteria [3, 4]. Thus, it is necessary to study how to improve immune responses and dysbiosis.

Probiotics are living organisms that are beneficial to the health of the host when ingested in moderate quantities [5, 6]. Lactic acid bacteria are representative probiotics that have various health-beneficial functions, such as normalization of intestinal flora and enhancement of immunity, as well as antioxidative and anti-inflammatory activities [7, 8]. Other studies [9, 10] have shown that indigestible food ingredients can help the growth of beneficial microorganisms in the intestine, especially probiotic strains, and improve human health. Thus, the effect of the combination of probiotics and prebiotics, called synbiotics, has been investigated on the basis of the hypothesis that synbiotics have better functional benefits than probiotics or prebiotics alone [11, 12].

*Lactobacillus gasseri* is a facultative anaerobic bacterium that is abundant in human and animal gastrointestinal tracts [13, 14]. They are used in diverse fermented dairy products and probiotics because they have immunomodulatory, antibacterial, and antihypertensive activities [15, 16]. *Cudrania tricuspidata* (CT) has been used in Asia as a medicine for antibacterial and anticancer therapy [17, 18] and was found to have a prebiotic effect by promoting the growth of beneficial bacteria in the intestine through its noncarbohydrate components comprising polyphenols [19].

Therefore, the objective of this study was to examine the effects of probiotics (*L. gasseri*), synbiotics (*L. gasseri*+CT extract), and peptides separated from the synbiotics on IBD improvements.

## 2. Materials and Methods

### 2.1. Animal

Five-week-old male C57BL/6 mice were purchased from Orient Bio Inc. (Seongnam, Gyeonggi, Korea) and adapted at 20-30°C and 40-60% humidity for a week. The mice were then divided into six experimental groups, as presented in Table 1. The animal experiment (SMWU-IACUC-1709-020) was approved by the Institute of Animal Care and Use Committee at Sookmyung Women's University.

### 2.2. Induction of IBD and Treatment

Mice were treated with 1-2% dextran sulfate sodium (DSS; MP Biomedicals Korea, Songpa-gu, Seoul, Korea) via the drinking water to induce IBD for 7 days; these mice comprised the IBD group. The negative control group received normal drinking water. Starting from day 0, the negative control mice were orally administered with phosphate-buffered saline (PBS: pH 7.4; 0.2 g KH_2_PO_4_, 1.5 g Na_2_HPO_4_·7H_2_O, 8.0 g NaCl, and 0.2 g KCl, in 1 L distilled water) using feeding needles once a day for 7 days. The IBD group was further divided in the following subgroups: the IBD-PBS group—orally administered with PBS; the IBD-Pro group—administered with probiotics (*L. gasseri* 505; Oh et al., 2016); the IBD-Syn group—administered with synbiotics (fermented powder of CT extract with *L. gasseri* 505); the IBD-Pep 1 group—administered with Pep 1; and the IBD-Pep 2 group—administered with Pep 2. Administration took place once a day for 7 days using feeding needles, as shown in Table 1. The oral administration continued for 4 more days without the DSS in the drinking water (Table 1). The two peptides Pep 1 and Pep 2, separated from the synbiotics as shown in Table 2 [19], were provided by Seoul Milk Central Research Institute (Ansan-si, Gyeonggi, Korea). The mice were fasted for 18 h and anesthetized with isoflurane (Hana pharm Co., Ltd., Hwaseong, Gyeonggi, Korea) for sacrifice. The intestines were rapidly removed, and the length of the small and large intestines was measured. They were then stored at -70°C. Parts of the large intestine tissues were fixed in 10% formalin solution (HISKO, Gunpo-si, Korea) for histopathological analysis.

### 2.3. Measurement of Body Weight and Observation of Fecal Conditions

The body weight of the mice was measured every 2 days. The disease activity index (DAI) scores were graded by observing the viscosity of feces and the presence or absence of blood in feces for 11 days from the first day of DSS water administration. The value was calculated according to the Hamers et al. [20].

### 2.4. Measurement of Nitric Oxide and Inflammatory Cytokine Concentrations in Serum

The blood samples were centrifuged at 94*×g* for 10 min to separate the serum. The serum (80 *μ*l) and the same volume of Griess reagent (Promega, Madison, WI, USA) were added to a 96-well plate and then incubated at room temperature for 10 min. The absorbance was measured with the Take3 system in Epoch™ Microplate Spectrophotometer (BioTek Instruments, Inc., Winooski, VT, USA) at 540 nm, and the concentrations of NO were calculated based on the quantitative curve of NaNO_2_ standard solution. In addition, the concentrations of inflammatory cytokines productions in the serum were measured using the ProcartaPlex™ mouse Th1/Th2 cytokine panel (11 plex) (Thermo Fisher Scientific, Waltham, MA, USA).

### 2.5. Measurement of Proinflammatory Cytokine mRNA Levels in the Large Intestine Tissue

Total mRNA was extracted from large intestine tissues using the RNeasy Mini Kit (Qiagen, Hilden, Germany) according to the manufacturer's instructions. The mRNA of large intestine tissues was quantified using the Take3 system in Epoch™ Microplate Spectrophotometer (BioTek Instruments). For quantitative real-time reverse transcription polymerase chain reaction (qRT-PCR) analysis, cDNA was synthesized using QuantiTect Reverse Transcription Kit (Qiagen) according to the manufacturer's instructions. The primers used in qRT-PCR for IL-1*β*, IL-6, and COX-2 are listed in Table 3, and the primers for TNF-*α* were purchased from Qiagen (Mm_Tnf_1_SG QuantiTect Primer Assay, Cat. no. QT00104006). qRT-PCR was performed on a Rotor-Gene Q instrument (Qiagen), using a Rotor-Gene SYBR® Green PCR Kit (Qiagen) according to the manufacturer's instructions. Relative fold changes of gene expression levels were analyzed using the −2^*ΔΔ*Ct^ method [24].

### 2.6. Histopathological Analysis of the Large Intestine Tissue

The fixed large intestine tissue samples were washed with distilled water and dehydrated using alcohol. They were fixed with paraffin, and these samples were then placed on slides and stained with hematoxylin and eosin (H&E). Histopathological analysis was performed at 200 magnification. The results were determined by referring to the international harmonization of nomenclature and diagnostic criteria (INHAND) standard, and the specimen lesions were compared.

### 2.7. Statistical Analysis

Experimental data were analyzed using the general linear procedure of SAS® version 9.4 (SAS Institute, Inc., Cary, NC, USA). The least square mean comparisons among the treatment groups were performed by a pairwise *t*-test at *α* = 0.05.

## 3. Results

### 3.1. Effects of Peptides on Mouse Weight and DAI

After 4 days of oral administration of 1% DSS, no obvious changes in fecal state, such as the presence of blood and diarrhea, were then observed. Thus, the DSS concentration was increased to 2% for 3 days, and bloody stool and diarrhea were observed. The body weight of the group treated with 2% DSS (IBD group) decreased, while that of the negative control group increased steadily (Table 4). The DAI scores were calculated using the fecal conditions (Table 5). As a result, the DAI scores for the negative control and the IBD group were 0 and 2, respectively (Table 6). The body weights in the IBD-Pro, IBD-Syn, IBD-Pep 1, or IBD-Pep 2 groups were not obviously different from that in the IBD-PBS-positive control group. The DAI scores (0.5-1.5) of the IBD-Pro, IBD-Pep 1, and IBD-Pep 2 groups were generally lower than that of the IBD-PBS-positive control group (DAI = 2), especially for the IBD-Pep 2 group (DAI = 0.5) (Table 6). The DAI score of the IBD-Pep 2 group was very close to that of the negative control (Table 6). Therefore, the Pep 2 (*α*_s2_-casein) separated from synbiotics could improve intestinal inflammation.

### 3.2. Comparisons of Small and Large Intestine Lengths

Yoon et al. [25] showed that the lengths of the small and large intestines tended to be shorter in mice with IBD. Similarly, we found that the lengths of the small and large intestines were shorter in the IBD-PBS-positive control, IBD-Pro, IBD-Syn, IBD-Pep 1, and IBD-Pep 2 groups than those in the negative control group, and the lengths of the large intestines in the IBD-PBS-positive control group were the shortest (Table 7).

### 3.3. Nitric Oxide and Inflammatory Cytokine Levels in Serum

**Figure 1:**
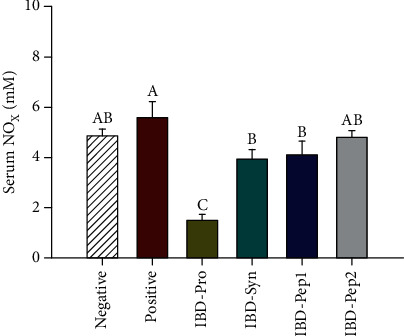
Serum nitric oxide (NO) concentration. ^A,B^Values are significantly different (*p* < 0.05).

**Figure 2:**
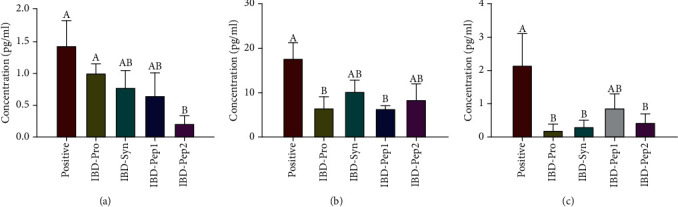
Inflammatory cytokine levels in the serum: (a) IFN-*γ*, (b) IL-6, and (c) TNF-*α*. ^A,B^Values are significantly different (*p* < 0.05).

The NO level was the highest in the positive control group, but the NO levels of the groups treated with IBD-Pro, IBD-Syn, and IBD-Pep 1, were significantly lower (*p* < 0.05) than that of the positive control (Figure 1). It indicates that the levels of inflammation were improved, when probiotics, synbiotics, and Pep1 were treated to the mice having intestinal inflammation. In serum inflammatory cytokines (IFN-*γ*, IL-6, and TNF-*α*), these cytokines were less secreted in all treatment groups, compared to the positive control (Figure 2). In particular, IFN-*γ* was lower (*p* < 0.05) in the IBD-Pep 2 group, and IL-6 was lower (*p* < 0.05) in the IBD-Pro and IBD-Pep 1 groups than those of the positive group. TNF-*α* was significantly lower (*p* < 0.05) in the IBD-Pro, IBD-Syn, and IBD-Pep 2 groups than that of the positive group (Figure 2).

### 3.4. mRNA Levels of Inflammatory Cytokine in Large Intestine Tissue

**Figure 3:**
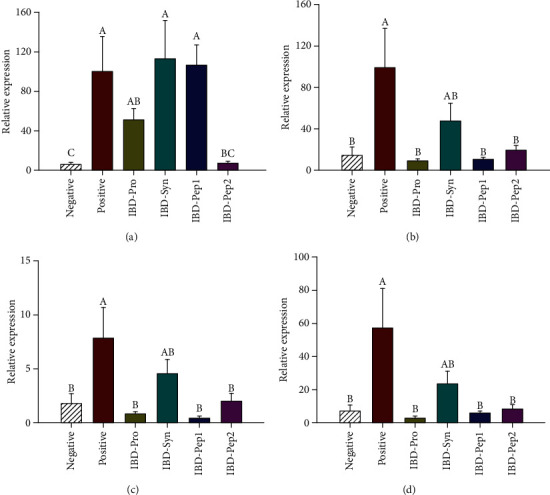
Inflammatory cytokine mRNA levels in the large intestine tissue: (a) IL-1*β*, (b) IL-6, (c) TNF-*α*, and (d) COX-2. ^A,B,C^Values are significantly different (*p* < 0.05).

Many studies have reported that the expression levels of IL-1*β* and TNF-*α* are increased in IBD patients [26]. We evaluated the mRNA levels of the proinflammatory cytokines such as IL-1*β*, IL-6, TNF-*α*, and COX-2 in the intestine of DSS-treated mice (IBD group), and the levels in the large intestine were increased (*p* < 0.05), indicating that DSS treatment obviously induced IBD (Figure 3). The mRNA levels of these inflammatory markers in large intestine tissues were significantly decreased (*p* < 0.05) in the IBD-Pep 2 group, compared to the IBD-PBS-positive control group. In addition, the mRNA levels of IL-6, TNF-*α*, and COX-2, but not IL-1*β*, were significantly decreased in the IBD-Pro and IBD-Pep 1 groups (*p* < 0.05) (Figure 3). Therefore, probiotics, Pep 1, and Pep 2 are efficient in inhibiting proinflammatory cytokine expression.

### 3.5. Histopathological Features of Large Intestine Tissue

**Figure 4:**
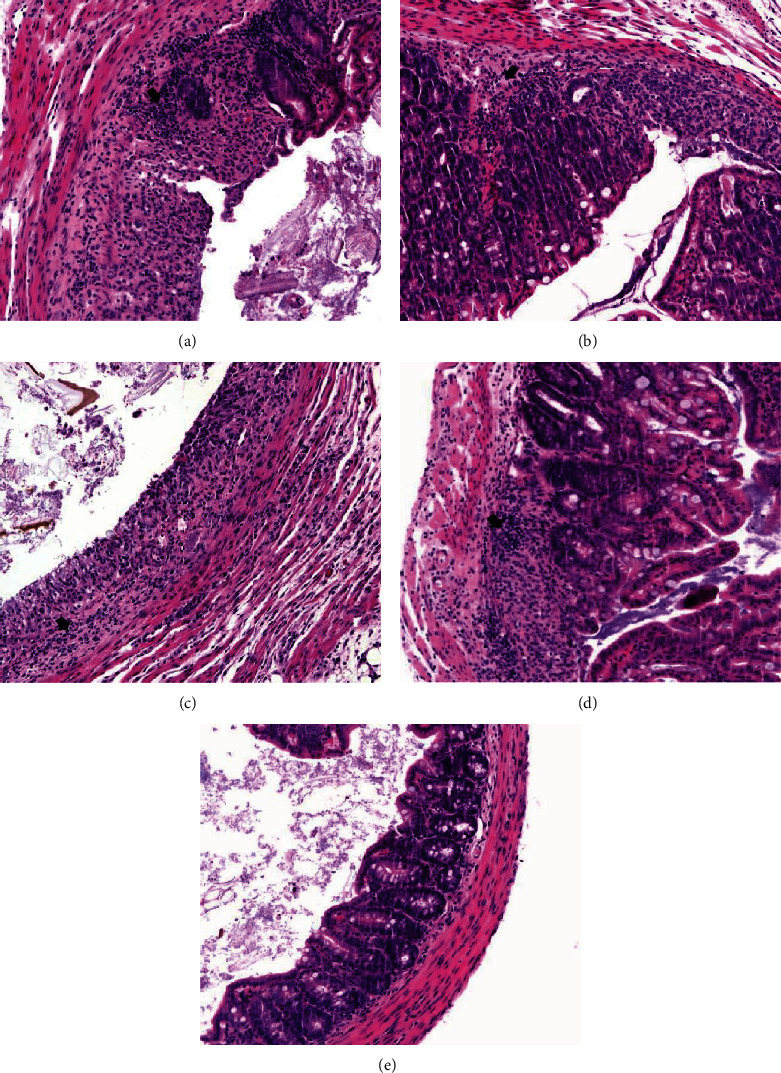
Histopathological features of jejunum of the IBD group of mice treated with PBS (a; IBD-PBS-positive group), probiotics (b; IBD-Pro), synbiotics (c; IBD-Syn), peptide 1 (d; IBD-Pep 1), and peptide 2 (e; IBD-Pep 2). Hematoxylin and eosin- (H&E-) stained sections of a mouse large intestine are shown. Black arrows indicate inflammation of the colitis mucosa. Magnifications: ×200.

IBD was mainly observed in the large intestine, and thus, histopathological features of jejunum tissue were examined. Jejunum inflammation was found in the IBD-PBS-positive control, IBD-Pro, IBD-Syn, and IBD-Pep 1 groups, compared to the negative control group (Figure 4). The degree of lesions was obviously lower in the IBD-Pep 2 treatment group than those in the IBD-PBS-positive control group and the other treatment groups (Figure 4). Therefore, *α*_s2_-casein (Pep 2) treatment results in the reduction of colorectal lesions in IBD mice.

## 4. Discussions

Bauer et al. [27] have suggested that DSS-induced IBD mice are characterized by weight loss, and Okayasu et al. [28] have suggested that weight loss and bloody stool are hallmarks of IBD. According to these studies, our results indicate that IBD was induced in the mice by 2% DSS treatment (Table 4), and the length of the small and large intestines became shorter by 2% DSS treatment, which is found in IBD patients [29] The DAI scores were also low in the IBD-Pep 1 and IBD-Pep 2 treatment groups, indicating that the inflammation was improved by Pep 1 and Pep 2 (Table 6).

NO level in serum is used as an important biomarker for active IBD patients [30]. When the inflammatory reaction of the colon occurs, the productions of cytokines such as TNF-*α* and IFN-*γ* induced third isoform inducible (iNOS) to increase the production of NO. TNF-*α* and IL-6 in the serum are related to intestinal disease, and all are upregulated in IBD patients [31]. Increased TNF-*α* due to intestinal disease stimulates the secretion of IFN-*γ* in IBD patients [32]. In our study, the inflammatory response was alleviated when the probiotics, synbiotics, and Pep 1 were orally administrated to mice which had the IBD. TNF-*α*, IFN-*γ*, and IL-6 secretions were generally lowered (Figure 2) by probiotics, Pep 1, and Pep 2 treatments. This result indicates that the treatments of probiotics, synbiotics, Pep 1, and Pep 2 can reduce NO and the inflammatory cytokines in serum.

Colonic lesions occur in IBD patients, and inflammatory cells infiltrate the lesion areas to produce proinflammatory cytokines [33]. However, the use of probiotics downregulates the expression of proinflammatory cytokines, limiting inflammatory reactions such as immune cell infiltration of mucous membranes [34]. Thus, the mRNA levels of proinflammatory cytokines such as IL-1*β*, IL-6, TNF-*α*, and COX-2 in the large intestine tissue were also measured in our study. As a result, the proinflammatory cytokines in the IBD-Pro, IBD-Pep 1, and IBD-Pep 2 were significantly reduced, compared to positive control. Also, in the histopathology results, the level of lesion in the jejunum was clearly reduced, especially in the IBD-Pep 2 group. This result indicates that probiotics, Pep 1, and Pep 2 reduce the inflammatory cytokine levels and Pep 2 reduces the level of lesion.

## 5. Conclusions

In conclusion, Pep 2, which contains the *α*_s2_-casein sequence of VYQHQKAMKPWIQPKTKVIPYVRYL separated from the synbiotics (fermented *CT* extract with *L. gasseri* 505), was commonly involved to improve the intestinal inflammation factors such as the DAI score, proinflammatory cytokine levels, and histopathological result. Therefore, Pep 2 could be used as a supplement for improving inflammation in IBD patients. However, a human application study should be conducted to confirm this effect.

## Figures and Tables

**Table 1:** Experimental groups.

Groups	Drinking water	Oral injection	*n*
Negative control	General water	PBS	8
IBD-PBS-positive control	DSS	PBS	8
IBD-Pro	DSS	Probiotics^1^	8
IBD-Syn	DSS	Synbiotics^2^	8
IBD-Pep 1	DSS	Peptide1^3^	8
IBD-Pep 2	DSS	Peptide2^4^	8

^1^Using *Lactobacillus gasseri* 505 injected in mice at a concentration of 10^8^ CFU/kg bw/day. ^2^Preparation of fermentation powders by using *Lactobacillus gasseri* 505 and *Cudrania tricuspidata* extract injected in mice at a concentration of 1500 mg/kg. ^3,4^Peptides isolated from fermentation powders injected in mice at a concentration of 20 mg/kg.

**Table 2:** Peptide information.

Groups	*m*/*z* peptide	Protein	Sequence	Reference
Pep 1	2479.2	*β*-casein	LSQSKVLPVPQKAVPYPQRDMP	[19]
Pep 2	3115.4	*α*_s2_-casein	VYQHQKAMKPWIQPKTKVIPYVRYL	

**Table 3:** Primers for quantitative real-time reverse transcription polymerase chain reaction.

Primer	Sequence (5′ to 3′)	Reference
Cytokine related primers		
IL-1*β*	F: AAC CTG CTG GTG TGT GAC GTT C	[21]
	R: CAG CAC GAG GCT TTT TTG TTG T	
IL-6	F: ACC AGA GGA AAT TTT GAA TAG GC	[22]
	R: TGA TGC ACT TGC AGA AAA CA	
COX-2	F: TGT ATC CCC CCA CAG TCA AAG ACA C	[23]
	R: GTG CTC CCG AAG CCA GAT GG	

**Table 4:** Weight change by dextran sulfate sodium autogenous feeding.

Groups	Initial weight (g)	Weight before sacrifice (g)
Negative control	21.5 ± 0.6^A,a^	22.3 ± 0.8^A,b^
IBD-PBS-positive control	21.3 ± 1.0^A,a^	15.4 ± 1.4^C,b^
IBD-Pro	21.2 ± 1.0^A,a^	17.4 ± 1.3^B,b^
IBD-Syn	21.4 ± 1.5^A,a^	16.0 ± 1.0^C,b^
IBD-Pep 1	21.1 ± 0.6^A,a^	15.0 ± 0.6^C,b^
IBD-Pep 2	20.9 ± 0.6^A,a^	16.3 ± 1.2^B,C,b^

^A,B,C^Values within the same column with different superscript letters are significantly different (*p* < 0.05). ^a,b^Values within the same row with different superscript letters are significantly different (*p* < 0.05).

**Table 5:** Scoring table for disease activity index.

Symptoms/score	Characteristics
Stool consistency	
0	Normal feces
1	Loose stool
2	Watery diarrhea
3	Slimy diarrhea, little blood
4	Severe watery diarrhea with blood
Blood in stool	
0	Negative
2	Positive
4	Gross bleeding

**Table 6:** Measurement of disease activity index using feces conditions the day after dextran sulfate sodium stops supplying and before sacrifice.

Groups	DAI scores	
	The day after DSS supply stopped	Before sacrifice
Negative control	0	0
IBD-PBS-positive control	3.5	2
IBD-Pro	2	1.5
IBD-Syn	2	2
IBD-Pep 1	4	1
IBD-Pep 2	2.5	0.5

**Table 7:** Differences in small and large intestine lengths among the groups (unit: cm).

Treatment	Small intestine	Large intestine
Negative control	38.24 ± 1.55^A^	7.37 ± 0.75^A^
IBD-PBS positive control	36.54 ± 1.15^B^	4.73 ± 0.53^C^
IBD-Pro	36.03 ± 1.34^B^	5.38 ± 0.57^B,C^
IBD-Syn	36.51 ± 1.45^B^	5.09 ± 0.54^B,C^
IBD-Pep 1	35.25 ± 1.21^B^	4.75 ± 0.33^B,C^
IBD-Pep 2	37.39 ± 1.78^A,B^	5.43 ± 0.68^B^

^A,B,C^Values within the same column with different superscript letters are significantly different (*p* < 0.05).

## Data Availability

The data used to support the findings of this study are available from the corresponding author upon request.
